# 1,4,8,11-Tetra­azoniacyclo­tetra­decane tetra­chloridocobaltate(II) dichloride

**DOI:** 10.1107/S1600536810025079

**Published:** 2010-07-03

**Authors:** Tarek Ferchichi, Besma Trojett, Hassouna Dhaouadi, Houda Marouani

**Affiliations:** a Laboratoire des Matériaux Utiles, Institut National de Recherche et d’Analyse Physico-chimique, Pole Technologique de Sidi-Thabet, 2020 Tunis, Tunisia; b Laboratoire de Chimie des Matériaux, Faculté des Sciences de Bizerte, 7021 Zarzouna Bizerte, Tunisia

## Abstract

The asymmetric unit of the title compound, (C_10_H_28_N_4_)[CoCl_4_]Cl_2_, contains two half-mol­ecules of the macrocycle, which are both completed by crystallographic inversion symmetry. In the dianion, the Co^2+^ cation is tetra­hedrally coordinated by four Cl atoms; the Co—Cl bond lengths correlate with the number of hydrogen bonds that the chloride ions accept. The crystal cohesion is supported by electrostatic inter­actions which, together with numerous N—H⋯Cl, N—H⋯(Cl,Cl) and C—H⋯Cl hydrogen bonds, lead to a three-dimensional network.

## Related literature

For background to organic–inorganic hybrid networks and their properties, see: Bu *et al.* (2001 ▶); Mitzi *et al.* (1999 ▶). For hydrogen-bonding in supra­molecular networks, see: Brammer *et al.* (2002 ▶). For related structures, see: El Glaoui *et al.* (2009 ▶); Jakubas *et al.* (2005 ▶); Adamski *et al.* (2009 ▶); Boyd & McFadyen (2007 ▶); Hashizume *et al.* (1999 ▶). 
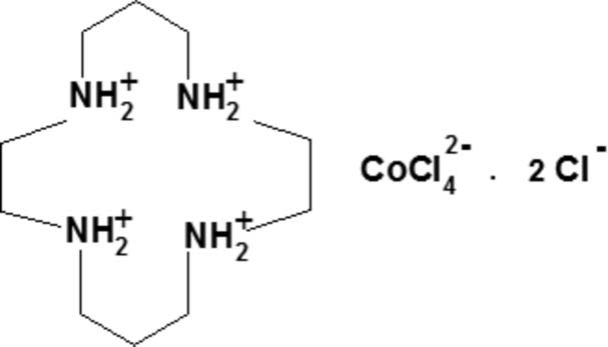


## Experimental

### 

#### Crystal data

(C_10_H_28_N_4_)[CoCl_4_]Cl_2_
*M* *_r_* = 475.99
Triclinic, 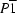

*a* = 7.4058 (10) Å
*b* = 8.1244 (10) Å
*c* = 17.147 (1) Å
α = 84.36 (2)°
β = 85.56 (2)°
γ = 77.84 (2)°
*V* = 1001.97 (19) Å^3^
*Z* = 2
Mo *K*α radiation
μ = 1.66 mm^−1^
*T* = 293 K
0.20 × 0.15 × 0.10 mm

#### Data collection

Enraf–Nonius CAD-4 diffractometer
21712 measured reflections
4375 independent reflections
4023 reflections with *I* > 2σ(*I*)
*R*_int_ = 0.012
2 standard reflections every 120 min  intensity decay: 1%

#### Refinement

*R*[*F* ^2^ > 2σ(*F* ^2^)] = 0.037
*wR*(*F* ^2^) = 0.098
*S* = 1.13
4375 reflections
191 parameters
H-atom parameters constrained
Δρ_max_ = 0.99 e Å^−3^
Δρ_min_ = −0.70 e Å^−3^

### 

Data collection: *CAD-4 EXPRESS* (Enraf–Nonius, 1994 ▶); cell refinement: *CAD-4 EXPRESS*; data reduction: *XCAD4* (Harms & Wocadlo, 1995 ▶); program(s) used to solve structure: *SHELXS97* (Sheldrick, 2008 ▶); program(s) used to refine structure: *SHELXL97* (Sheldrick, 2008 ▶); molecular graphics: *ORTEP-3* (Farrugia, 1997 ▶); software used to prepare material for publication: *WinGX* (Farrugia, 1999 ▶).

## Supplementary Material

Crystal structure: contains datablocks I, global. DOI: 10.1107/S1600536810025079/hb5513sup1.cif

Structure factors: contains datablocks I. DOI: 10.1107/S1600536810025079/hb5513Isup2.hkl

Additional supplementary materials:  crystallographic information; 3D view; checkCIF report

## Figures and Tables

**Table 1:** Selected bond lengths (Å)

Co—Cl1	2.2609 (8)
Co—Cl2	2.2950 (9)
Co—Cl3	2.2963 (7)
Co—Cl4	2.3170 (8)

**Table 2:** Hydrogen-bond geometry (Å, °)

*D*—H⋯*A*	*D*—H	H⋯*A*	*D*⋯*A*	*D*—H⋯*A*
N1—H1*A*⋯Cl6	0.90	2.26	3.155 (2)	171
N1—H1*B*⋯Cl4^i^	0.90	2.65	3.370 (2)	138
N1—H1*B*⋯Cl2	0.90	2.75	3.315 (2)	122
N2—H2*A*⋯Cl6	0.90	2.20	3.090 (2)	170
N2—H2*B*⋯Cl2^ii^	0.90	2.51	3.284 (2)	144
N2—H2*B*⋯Cl4^ii^	0.90	2.95	3.609 (2)	131
N3—H3*A*⋯Cl5^iii^	0.90	2.27	3.129 (2)	160
N3—H3*B*⋯Cl5^i^	0.90	2.32	3.132 (2)	151
N4—H4*A*⋯Cl4	0.90	2.50	3.298 (2)	147
N4—H4*A*⋯Cl2^iv^	0.90	2.93	3.508 (2)	123
N4—H4*B*⋯Cl5	0.90	2.43	3.192 (2)	143
C2—H2*C*⋯Cl6^ii^	0.97	2.74	3.589 (3)	147
C6—H6*B*⋯Cl3^v^	0.97	2.80	3.758 (3)	169
C10—H10*A*⋯Cl3^v^	0.97	2.92	3.820 (3)	155
C3—H3*D*⋯Cl1^vi^	0.97	2.74	3.610 (3)	150
C3—H3*C*⋯Cl6^vi^	0.97	2.79	3.634 (3)	147

Symmetry codes: (i) 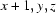; (ii) 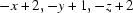; (iii) 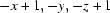; (iv) 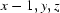; (v) 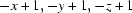; (vi) 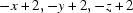.

**Fig. 1.**
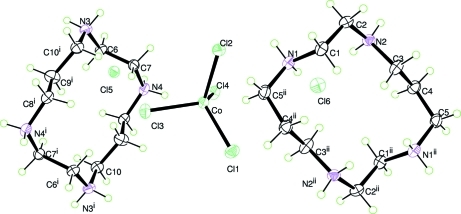
The molecular structure of (I): displacement ellipsoids are drawn at the 30% probability level. H atoms are represented as spheres of arbitrary radius. [Symmetry code: (i) (-x + 1, -y + 1, -z + 1))(ii) (x, -1 + y, z)].

**Fig. 2.**
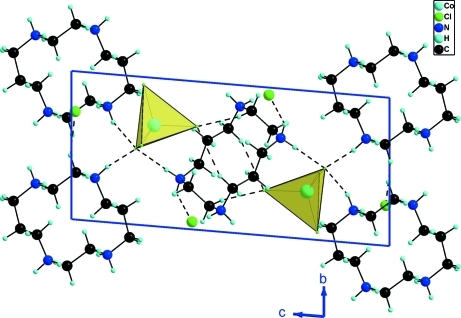
Projection of (I) along the a axis.

## supplementary crystallographic information

### Comment

The rational design and synthesis of organic-inorganic hybrid materials have attracted an increasing interest in recent years not only from a structural point of view, but also due to their potential applications in different areas such as catalysis, medicine, electrical conductivity, magnetism and photochemistry (e.g. Bu *et al.*, 2001).

A large number of transition metal when associated to organic molecule which presents potential sites of the hydrogen bonding interactions, exhibit interesting one- (1-D), two- (2-D), and three-dimensional (3-D) structures. These organic molecules may be present different and interesting properties which can profoundly influence the structures of inorganic component in the resultant hybrid material. Such organic-inorganic hybrid materials can combine appropriate characteristics of each component to produce novel structural types, as well as new properties arising from the interplay of the two components (Mitzi *et al.*, 1999).

The asymmetric unit of the [CoCl_4_](C_10_H_28_N_4_),2Cl, (I). contains one tetrachlorocobalt anion, one organic cation and two chloride anion as shown in Fig. 1. The cohesion and the stability between these different components are assured by the network hydrogen bonding of type (N—H···Cl). However, the energetic of N—H···Cl—*M* (*M* = metal) hydrogen bonds and their possible roles in supramolecular chemistry have only been recently described in details (Brammer *et al.*, 2002). This type of hydrogen bond is also observed in other hybrid compounds such as Bis(5-Chloro-2,4-Dimethoxyanilinium) Tetrachlorozincate Trihydrate (El Glaoui *et al.*, 2009) and pyrrolidinium hexachloroantimonate (V) (Jakubas *et al.*, 2005).

The Co^2+^ entity is tetrahedrally coordinated to four chloride atoms as shown in Figure 2. The distortion from the ideal geometry is small. This situation is also observed in others compounds which contain CoCl_4_^2-^ entity as an anion (Adamski *et al.*, 2009). Examination of the CoCl_4_^2-^ geometry shows two types of Co—Cl distances. The largest ones 2.3170 (8) Å, 2.2963 (7) Å and 2.2950 (9) Å, while the smallest one is 2.2609 (8) Å. The average values of the Co—Cl distances and Cl—Co—Cl angles are 2.2923 Å and 109.52°, respectively. These geometrical features have also been noticed in others crystal structure (Boyd *et al.*, 2007); (Hashizume *et al.*, 1999).

The differences in the Co—Cl bond lengths correlate with the number of hydrogen bonds accepted by the Cl atom: Co—Cl4 bond is the longest (2.3170 (8) Å); Cl4 accepts three H-bonds, Co—Cl2 and Co—Cl3 have similar, intermediate lengths, and Cl1, which accepts only C—H···Cl hydrogen bond, makes the shortest Co—Cl bond.

The four N atoms of the macrocyclic ring are protonated, which provide the cations as formula (C_10_H_28_N_4_)^4+^, for neutralize the negative charge of the anionic part. Crystal cohesion and stability are supported by electrostatic interactions which, together with N—H···Cl and C—H···Cl hydrogen bonds, build up a three-dimensional network.

### Experimental

The hexahydrate of chloride cobalt (II) CoCl_2_.6H_2_O (3.1 mmol) was added to an aqueous solution containing a stochiometric ratio of C_10_H_24_N_4_ (1,4,8,11-tetraazacyclotetradecane) (3.1 mmol) under continuous stirring. A pink precipitate was formed, which was completely dissolved by adding an aqueous solution of HCl until it disappeared. The obtained solution was slowly evaporated at room temperature for several days until the formation of blue prisms of (I). The synthesis is reproducible and crystals obtained in this way are stable for a long time under normal conditions of temperature and humidity.

### Crystal data

**Table:**

(C_10_H_28_N_4_)[CoCl_4_]Cl_2_	*Z* = 2
*M**_r_* = 475.99	*F*(000) = 490
Triclinic, *P*1	*D*_x_ = 1.578 Mg m^−^^3^
Hall symbol: -P 1	Mo *K*α radiation, λ = 0.71073 Å
*a* = 7.4058 (10) Å	Cell parameters from 25 reflections
*b* = 8.1244 (10) Å	θ = 12–15°
*c* = 17.147 (1) Å	µ = 1.66 mm^−^^1^
α = 84.36 (2)°	*T* = 293 K
β = 85.56 (2)°	Prism, blue
γ = 77.84 (2)°	0.20 × 0.15 × 0.10 mm
*V* = 1001.97 (19) Å^3^	

### Data collection

**Table:**

Enraf–Nonius CAD-4 diffractometer	*R*_int_ = 0.012
Radiation source: fine-focus sealed tube	θ_max_ = 27.0°, θ_min_ = 2.4°
graphite	*h* = −9→2
non–profiled ω/2θ scans	*k* = −10→10
21712 measured reflections	*l* = −21→21
4375 independent reflections	2 standard reflections every 120 min
4023 reflections with *I* > 2σ(*I*)	intensity decay: −1%

### Refinement

**Table:**

Refinement on *F*^2^	Secondary atom site location: difference Fourier map
Least-squares matrix: full	Hydrogen site location: inferred from neighbouring sites
*R*[*F*^2^ > 2σ(*F*^2^)] = 0.037	H-atom parameters constrained
*wR*(*F*^2^) = 0.098	*w* = 1/[σ^2^(*F*_o_^2^) + (0.0484*P*)^2^ + 1.0784*P*] where *P* = (*F*_o_^2^ + 2*F*_c_^2^)/3
*S* = 1.13	(Δ/σ)_max_ = 0.001
4375 reflections	Δρ_max_ = 0.99 e Å^−^^3^
191 parameters	Δρ_min_ = −0.69 e Å^−^^3^
0 restraints	Extinction correction: *SHELXL97* (Sheldrick, 2008), Fc^*^=kFc[1+0.001xFc^2^λ^3^/sin(2θ)]^-1/4^
Primary atom site location: structure-invariant direct methods	Extinction coefficient: 0.0098 (15)

### Special details

**Table:**

Geometry. All e.s.d.'s (except the e.s.d. in the dihedral angle between two l.s. planes) are estimated using the full covariance matrix. The cell e.s.d.'s are taken into account individually in the estimation of e.s.d.'s in distances, angles and torsion angles; correlations between e.s.d.'s in cell parameters are only used when they are defined by crystal symmetry. An approximate (isotropic) treatment of cell e.s.d.'s is used for estimating e.s.d.'s involving l.s. planes.
Refinement. Refinement of *F*^2^ against ALL reflections. The weighted *R*-factor *wR* and goodness of fit *S* are based on *F*^2^, conventional *R*-factors *R* are based on *F*, with *F* set to zero for negative *F*^2^. The threshold expression of *F*^2^ > σ(*F*^2^) is used only for calculating *R*-factors(gt) *etc*. and is not relevant to the choice of reflections for refinement. *R*-factors based on *F*^2^ are statistically about twice as large as those based on *F*, and *R*- factors based on ALL data will be even larger.

### Fractional atomic coordinates and isotropic or equivalent isotropic displacement parameters (Å^2^)

**Table:**

	*x*	*y*	*z*	*U*_iso_*/*U*_eq_	
Co	0.70021 (4)	0.68008 (4)	0.739869 (18)	0.02545 (12)	
Cl1	0.59983 (10)	0.94777 (8)	0.77340 (4)	0.03784 (17)	
Cl2	0.98382 (9)	0.53726 (8)	0.77981 (4)	0.03170 (15)	
Cl3	0.72293 (9)	0.69599 (9)	0.60513 (3)	0.03302 (16)	
Cl4	0.49963 (9)	0.51312 (8)	0.79699 (4)	0.03371 (16)	
Cl5	0.13290 (9)	0.05084 (8)	0.62097 (4)	0.03219 (15)	
Cl6	0.79324 (9)	0.72834 (9)	0.98838 (4)	0.03847 (17)	
N4	0.3477 (3)	0.3336 (3)	0.65786 (12)	0.0285 (4)	
H4A	0.3385	0.3878	0.7018	0.034*	
H4B	0.3043	0.2383	0.6705	0.034*	
N1	1.1540 (3)	0.7804 (3)	0.88701 (12)	0.0287 (4)	
H1A	1.0461	0.7781	0.9147	0.034*	
H1B	1.1948	0.6781	0.8688	0.034*	
N2	1.1506 (3)	0.7095 (2)	1.07369 (12)	0.0260 (4)	
H2A	1.0476	0.7014	1.0510	0.031*	
H2B	1.1708	0.6263	1.1126	0.031*	
N3	0.7691 (3)	0.1442 (3)	0.53021 (13)	0.0288 (4)	
H3A	0.7801	0.0709	0.4932	0.035*	
H3B	0.8470	0.0955	0.5675	0.035*	
C5	0.8823 (4)	1.0892 (3)	1.18244 (15)	0.0317 (5)	
H5A	0.7709	1.0990	1.2168	0.038*	
H5B	0.9785	1.1149	1.2117	0.038*	
C7	0.5487 (3)	0.2847 (3)	0.63236 (14)	0.0266 (5)	
H7A	0.6180	0.2286	0.6766	0.032*	
H7B	0.5958	0.3855	0.6142	0.032*	
C2	1.3114 (3)	0.6829 (3)	1.01371 (15)	0.0293 (5)	
H2C	1.3274	0.5700	0.9968	0.035*	
H2D	1.4227	0.6890	1.0387	0.035*	
C8	0.2242 (3)	0.4440 (3)	0.59907 (15)	0.0282 (5)	
H8A	0.0990	0.4699	0.6224	0.034*	
H8B	0.2223	0.3819	0.5537	0.034*	
C1	1.2914 (3)	0.8090 (3)	0.94166 (14)	0.0276 (5)	
H1C	1.2531	0.9223	0.9585	0.033*	
H1D	1.4111	0.8016	0.9134	0.033*	
C4	0.9406 (4)	0.9072 (3)	1.16103 (15)	0.0308 (5)	
H4C	0.9587	0.8328	1.2089	0.037*	
H4D	0.8417	0.8787	1.1345	0.037*	
C6	0.5756 (3)	0.1671 (3)	0.56660 (15)	0.0274 (5)	
H6A	0.5508	0.0582	0.5874	0.033*	
H6B	0.4884	0.2139	0.5268	0.033*	
C10	0.1664 (3)	0.7006 (3)	0.50678 (16)	0.0297 (5)	
H10A	0.1674	0.6241	0.4667	0.036*	
H10B	0.0399	0.7333	0.5279	0.036*	
C3	1.1177 (4)	0.8759 (3)	1.10836 (17)	0.0336 (6)	
H3C	1.1104	0.9656	1.0663	0.040*	
H3D	1.2218	0.8794	1.1387	0.040*	
C9	0.2862 (4)	0.6085 (3)	0.57213 (18)	0.0371 (6)	
H9A	0.2739	0.6784	0.6157	0.045*	
H9B	0.4151	0.5849	0.5532	0.045*	

### Atomic displacement parameters (Å^2^)

**Table:**

	*U*^11^	*U*^22^	*U*^33^	*U*^12^	*U*^13^	*U*^23^
Co	0.02721 (19)	0.02537 (18)	0.02405 (18)	−0.00576 (13)	0.00061 (12)	−0.00424 (12)
Cl1	0.0455 (4)	0.0261 (3)	0.0424 (4)	−0.0072 (3)	−0.0007 (3)	−0.0074 (3)
Cl2	0.0307 (3)	0.0305 (3)	0.0328 (3)	−0.0039 (2)	−0.0015 (2)	−0.0029 (2)
Cl3	0.0324 (3)	0.0433 (4)	0.0248 (3)	−0.0102 (3)	−0.0001 (2)	−0.0059 (2)
Cl4	0.0350 (3)	0.0343 (3)	0.0348 (3)	−0.0141 (3)	0.0055 (2)	−0.0087 (2)
Cl5	0.0361 (3)	0.0301 (3)	0.0321 (3)	−0.0084 (2)	−0.0056 (2)	−0.0048 (2)
Cl6	0.0296 (3)	0.0405 (4)	0.0482 (4)	−0.0144 (3)	0.0001 (3)	−0.0041 (3)
N4	0.0340 (11)	0.0270 (10)	0.0257 (10)	−0.0094 (9)	0.0056 (8)	−0.0071 (8)
N1	0.0315 (11)	0.0259 (10)	0.0297 (10)	−0.0072 (8)	0.0032 (8)	−0.0080 (8)
N2	0.0289 (10)	0.0208 (9)	0.0271 (10)	−0.0036 (8)	0.0004 (8)	−0.0001 (8)
N3	0.0262 (10)	0.0248 (10)	0.0340 (11)	−0.0006 (8)	−0.0022 (8)	−0.0060 (8)
C5	0.0376 (14)	0.0331 (13)	0.0241 (11)	−0.0065 (11)	0.0034 (10)	−0.0066 (10)
C7	0.0286 (12)	0.0254 (11)	0.0263 (11)	−0.0059 (9)	−0.0027 (9)	−0.0030 (9)
C2	0.0253 (11)	0.0258 (11)	0.0351 (13)	−0.0011 (9)	0.0020 (10)	−0.0062 (10)
C8	0.0258 (11)	0.0281 (12)	0.0310 (12)	−0.0055 (9)	0.0033 (9)	−0.0076 (9)
C1	0.0256 (11)	0.0301 (12)	0.0288 (12)	−0.0094 (9)	0.0040 (9)	−0.0067 (9)
C4	0.0355 (13)	0.0282 (12)	0.0280 (12)	−0.0071 (10)	0.0053 (10)	−0.0030 (10)
C6	0.0262 (11)	0.0235 (11)	0.0328 (12)	−0.0051 (9)	0.0007 (9)	−0.0054 (9)
C10	0.0235 (11)	0.0314 (12)	0.0347 (13)	−0.0064 (9)	−0.0006 (10)	−0.0045 (10)
C3	0.0334 (13)	0.0319 (13)	0.0383 (14)	−0.0100 (11)	0.0063 (11)	−0.0151 (11)
C9	0.0392 (14)	0.0281 (13)	0.0475 (16)	−0.0122 (11)	−0.0130 (12)	−0.0003 (11)

### Geometric parameters (Å, °)

**Table:**

Co—Cl1	2.2609 (8)	C7—C6	1.523 (3)
Co—Cl2	2.2950 (9)	C7—H7A	0.9700
Co—Cl3	2.2963 (7)	C7—H7B	0.9700
Co—Cl4	2.3170 (8)	C2—C1	1.521 (4)
N4—C7	1.500 (3)	C2—H2C	0.9700
N4—C8	1.509 (3)	C2—H2D	0.9700
N4—H4A	0.9000	C8—C9	1.522 (4)
N4—H4B	0.9000	C8—H8A	0.9700
N1—C1	1.503 (3)	C8—H8B	0.9700
N1—C5^i^	1.515 (3)	C1—H1C	0.9700
N1—H1A	0.9000	C1—H1D	0.9700
N1—H1B	0.9000	C4—C3	1.523 (4)
N2—C3	1.495 (3)	C4—H4C	0.9700
N2—C2	1.506 (3)	C4—H4D	0.9700
N2—H2A	0.9000	C6—H6A	0.9700
N2—H2B	0.9000	C6—H6B	0.9700
N3—C6	1.500 (3)	C10—N3^ii^	1.506 (3)
N3—C10^ii^	1.506 (3)	C10—C9	1.521 (4)
N3—H3A	0.9000	C10—H10A	0.9700
N3—H3B	0.9000	C10—H10B	0.9700
C5—N1^i^	1.515 (3)	C3—H3C	0.9700
C5—C4	1.524 (4)	C3—H3D	0.9700
C5—H5A	0.9700	C9—H9A	0.9700
C5—H5B	0.9700	C9—H9B	0.9700
			
Cl1—Co—Cl2	117.71 (3)	N2—C2—H2D	108.6
Cl1—Co—Cl3	106.35 (3)	C1—C2—H2D	108.6
Cl2—Co—Cl3	106.05 (3)	H2C—C2—H2D	107.6
Cl1—Co—Cl4	109.41 (3)	N4—C8—C9	113.0 (2)
Cl2—Co—Cl4	103.43 (3)	N4—C8—H8A	109.0
Cl3—Co—Cl4	114.17 (3)	C9—C8—H8A	109.0
C7—N4—C8	116.34 (19)	N4—C8—H8B	109.0
C7—N4—H4A	108.2	C9—C8—H8B	109.0
C8—N4—H4A	108.2	H8A—C8—H8B	107.8
C7—N4—H4B	108.2	N1—C1—C2	113.2 (2)
C8—N4—H4B	108.2	N1—C1—H1C	108.9
H4A—N4—H4B	107.4	C2—C1—H1C	108.9
C1—N1—C5^i^	115.3 (2)	N1—C1—H1D	108.9
C1—N1—H1A	108.5	C2—C1—H1D	108.9
C5^i^—N1—H1A	108.5	H1C—C1—H1D	107.8
C1—N1—H1B	108.5	C3—C4—C5	113.2 (2)
C5^i^—N1—H1B	108.5	C3—C4—H4C	108.9
H1A—N1—H1B	107.5	C5—C4—H4C	108.9
C3—N2—C2	114.07 (19)	C3—C4—H4D	108.9
C3—N2—H2A	108.7	C5—C4—H4D	108.9
C2—N2—H2A	108.7	H4C—C4—H4D	107.7
C3—N2—H2B	108.7	N3—C6—C7	110.9 (2)
C2—N2—H2B	108.7	N3—C6—H6A	109.5
H2A—N2—H2B	107.6	C7—C6—H6A	109.5
C6—N3—C10^ii^	117.64 (19)	N3—C6—H6B	109.5
C6—N3—H3A	107.9	C7—C6—H6B	109.5
C10^ii^—N3—H3A	107.9	H6A—C6—H6B	108.1
C6—N3—H3B	107.9	N3^ii^—C10—C9	112.8 (2)
C10^ii^—N3—H3B	107.9	N3^ii^—C10—H10A	109.0
H3A—N3—H3B	107.2	C9—C10—H10A	109.0
N1^i^—C5—C4	114.7 (2)	N3^ii^—C10—H10B	109.0
N1^i^—C5—H5A	108.6	C9—C10—H10B	109.0
C4—C5—H5A	108.6	H10A—C10—H10B	107.8
N1^i^—C5—H5B	108.6	N2—C3—C4	112.5 (2)
C4—C5—H5B	108.6	N2—C3—H3C	109.1
H5A—C5—H5B	107.6	C4—C3—H3C	109.1
N4—C7—C6	110.50 (19)	N2—C3—H3D	109.1
N4—C7—H7A	109.6	C4—C3—H3D	109.1
C6—C7—H7A	109.6	H3C—C3—H3D	107.8
N4—C7—H7B	109.6	C10—C9—C8	108.7 (2)
C6—C7—H7B	109.6	C10—C9—H9A	109.9
H7A—C7—H7B	108.1	C8—C9—H9A	109.9
N2—C2—C1	114.7 (2)	C10—C9—H9B	109.9
N2—C2—H2C	108.6	C8—C9—H9B	109.9
C1—C2—H2C	108.6	H9A—C9—H9B	108.3

Symmetry codes: (i) −*x*+2, −*y*+2, −*z*+2; (ii) −*x*+1, −*y*+1, −*z*+1.

### Hydrogen-bond geometry (Å, °)

**Table:**

*D*—H···*A*	*D*—H	H···*A*	*D*···*A*	*D*—H···*A*
N1—H1A···Cl6	0.90	2.26	3.155 (2)	171
N1—H1B···Cl4^iii^	0.90	2.65	3.370 (2)	138
N1—H1B···Cl2	0.90	2.75	3.315 (2)	122
N2—H2A···Cl6	0.90	2.20	3.090 (2)	170
N2—H2B···Cl2^iv^	0.90	2.51	3.284 (2)	144
N2—H2B···Cl4^iv^	0.90	2.95	3.609 (2)	131
N3—H3A···Cl5^v^	0.90	2.27	3.129 (2)	160
N3—H3B···Cl5^iii^	0.90	2.32	3.132 (2)	151
N4—H4A···Cl4	0.90	2.50	3.298 (2)	147
N4—H4A···Cl2^vi^	0.90	2.93	3.508 (2)	123
N4—H4B···Cl5	0.90	2.43	3.192 (2)	143
C2—H2C···Cl6^iv^	0.97	2.74	3.589 (3)	147
C6—H6B···Cl3^ii^	0.97	2.80	3.758 (3)	169
C10—H10A···Cl3^ii^	0.97	2.92	3.820 (3)	155
C3—H3D···Cl1^i^	0.97	2.74	3.610 (3)	150
C3—H3C···Cl6^i^	0.97	2.79	3.634 (3)	147

Symmetry codes: (iii) *x*+1, *y*, *z*; (iv) −*x*+2, −*y*+1, −*z*+2; (v) −*x*+1, −*y*, −*z*+1; (vi) *x*−1, *y*, *z*; (ii) −*x*+1, −*y*+1, −*z*+1; (i) −*x*+2, −*y*+2, −*z*+2.
